# Atypical Cowpox Virus Infection in Smallpox-Vaccinated Patient, France

**DOI:** 10.3201/eid2502.171433

**Published:** 2019-02

**Authors:** Julien Andreani, Jean-Philippe Arnault, Jacques Y. Bou Khalil, Jônatas Abrahão, Enora Tomei, Emeline Vial, Marion Le Bideau, Didier Raoult, Bernard La Scola

**Affiliations:** Institut Hospitalo-Universitaire Méditerranée Infection, Marseille (J. Andreani, J.Y. Bou Khalil, E. Tomei, E. Vial, M. Le Bideau, D. Raoult, B. La Scola);; Aix-Marseille Université, Marseille, France (J. Andreani, J.Y. Bou Khalil, D. Raoult, B. La Scola);; Centre Hospitalier Universitaire Amiens-Picardie, Amiens, France (J.-P. Arnault);; Universidade Federal de Minas Gerais, Belo Horizonte, Brazil (J. Abrahão)

**Keywords:** cowpox virus, *Poxviridae*, human infection, route of infection, case report, France, viruses, zoonoses, smallpox, vaccination, variola virus

## Abstract

We report a case of atypical cowpox virus infection in France in 2016. The patient sought care for thoracic lesions after injury from the sharp end of a metallic guardrail previously stored in the ground. We isolated a cowpox virus from the lesions and sequenced its whole genome. The patient reported that he had been previously vaccinated against smallpox. We describe an alternative route of cowpox virus infection and raise questions about the immunological status of smallpox-vaccinated patients for circulating orthopoxviruses.

The genus *Orthopoxvirus* (family *Poxviridae*) is composed of 10 recognized viral species that infect vertebrates and cause serologic cross-reactions. Among the orthopoxviruses, variola virus, which causes smallpox in humans, was associated with the death of millions of persons. An extensive vaccination campaign promoted by the World Health Organization and using multiple vaccinia virus variants (*1*) during the 1960s and 1970s led to a declaration that smallpox was eradicated in 1980, and vaccination ceased. Most persons born after 1980 have not received smallpox vaccination, and so there is a reduced level of population-based immunity. Coincidentally or not, some zoonotic orthopoxvirus species are re-emerging in an increasing and alarming number of cases worldwide, including vaccinia virus in Brazil and India (*2*), monkeypox virus in Africa (*3*,*4*), cowpox virus in Europe and Asia (*5*), and novel orthopoxvirus-related strains in the United States (in Alaska and Georgia) (*6*–*8*). 

Cowpox virus infection in humans causes local cutaneous pustular affections, which may in some cases disseminate and become fatal in immunocompromised patients (*9*,*10*). Recent studies showed that cowpox virus is a unique name given to different strains with numerous misnomers (*11*–*13*). Rodents seem to be the main reservoirs of cowpox virus (*14*). Description of cowpox virus infections in cows has been rare in the last years (*15*). Because cowpox virus can infect a broad range of hosts, viral infections have been reported in cats, monkeys, elephants, llamas, and other vertebrates at zoos in Europe (*16*,*17*). Since the 2000s, cowpox virus infections in humans have been frequently associated with direct contact between patients and rodents (*18*–*21*), causing lesions mainly on the hands, arms, face, and neck. Human infection can also occur through intermediate hosts, notably by domestic cats, which are commonly infected with cowpox virus through contact with rodents (*22*). Although infection by fomites is not frequently described for cowpox virus, it is a well-described route of infection for other orthopoxviruses, such as vaccinia virus in Brazil (*23*). 

We report an atypical cowpox virus human infection in France in 2016, in which the patient had a pustular lesion on the laterothoracic area, but reported no direct contact with infected domestic or wild animals. We present our analysis of this novel viral strain, cowpox virus France Amiens 2016, describe its complete genome, review some morphological aspects of its infectious cycle, and discuss the probable way of transmission.

## Materials and Methods

### Clinical Examination and Disease Course

**Figure 1:**
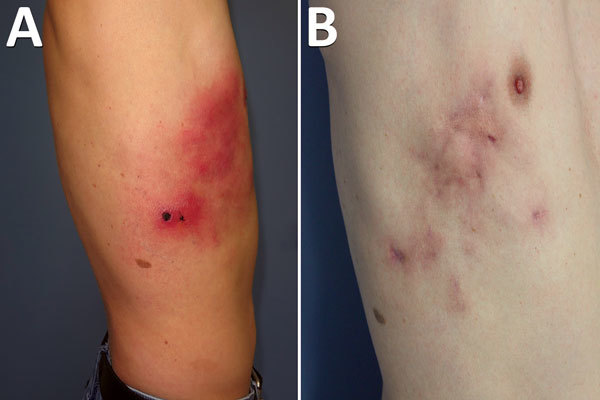
Cowpox virus infection in smallpox-vaccinated patient in France, 2016. A) Profile appearance of the patient’s torso 1 month after the initial trauma. B) Appearance 9 months after the initial trauma.

A 45-year-old man, an electrician, had a work accident and was injured by the sharp end of a metallic building site’s guardrail, which was stored in the ground. The lesion was superficial; it affected the derma with little bleeding and did not reach the hypoderma tissue. The laterothoracic wound did not heal and turned into a black eschar with painful cellulitis spreading to the front and upward on the laterothoracic area slowly over 4 weeks (Figure 1, panel A). Multiple treatments were administered by the patient’s general physician with no effect on the course of the disease: amoxicillin (1 g 2×/d), valaciclovir (1 g 3×/d), pristinamycin (1 g 2×/d), ceftriaxone (1 g 4×/d), and doxycycline (100 mg 2×/d).

After 4 weeks, the patient sought further care at Centre Hospitalier Universitaire (CHU) Amiens-Picardie in Amiens, France. He was apyretic, with good general condition and normal vital signs. The whole cellulitis was painful, associated with multiple subcutaneous abscesses and axillary adenopathies. Relevant biologic exams showed increased lymphocyte count (46%, 3.6 G/L); mild hepatitis (aspartate aminotransferase 1.5× the upper limit of normal [ULN], alanine-aminotransferase 1.5× ULN, γ-glutamyl transferase 1.5×ULN); and C-reactive protein (22 mg/L). Electrolytes, prothrombin ratio, partial thromboplastin time, hemoglobin, platelet count, creatinine, procalcitonin, and fibrinogen were normal. Skin biopsy showed a predominantly eosinophilic and neutrophilic necrotizing dermohypodermitis, with intravascular thrombi without vasculitis. Moreover, periodic acid–Schiff (PAS) and Grocott staining showed no pathogens.

At this time, a disease by inoculation was suspected. Results of routine skin biopsy cultures for fungi, bacteria, and mycobacteria were negative, as were intracellular cultures performed on the scar biopsy for *Ricketssia* spp. Results of molecular detection of herpesviruses, herpes virus 1/2, and varicella zoster virus were negative, as were *Bartonella henselae* and *Franciscela tularensis* serologic test results.

The apex of the disease occurred 8 weeks after the initial trauma. Cellulitis grew through the hemithoracic region with purulent discharge from open wounds because of severe delayed healing. The pain required morphine. No wound debridement was needed. Pain spontaneously ceased 4 months after the initial trauma, and the patient was declared healed after 9 months (Figure 1, panel B).

### Virus Detection, Isolation, and Production

Similar to the process Ninove et al. described in 2009 (*18*), a sample was sent to the Institut Hospitalo-Universitaire Méditerranée Infection, Marseille, France, diagnostic laboratory to explore intracellular microorganisms, especially *Rickettsia* spp. (intracellular bacteria), suspected by the presence of eschar. Nevertheless, we performed other PCR diagnostics at Centre Hospitalier Universitaire Amiens-Picardie. We performed biochemical, hematologic, and serologic examinations using Siemens analyzers (Siemens, https://www.healthcare.siemens.com). We used kits and reagents to detect *Bartonella* spp., *Bartonella henselae*, and *Bartonella quintana* (Eurobio indirect immunofluorescence assay, http://www.eurobio.fr) and the Virion ELISA classic kit (Serion Diagnostics, https://www.serion-diagnostics.de) to detect *Francisella tularensis*.

At Institut Hospitalo-Universitaire Méditerranée Infection, we performed PCR assays on the cutaneous biopsy taken from the pustular area when the sample was received. To detect orthopoxvirus, we used the primers F-5′-TGATGCAACTCTATCATGTARTCG, R-5′-CAAGACGTCGCTTTTRGCAG, and 6FAM- TGCTTGGTATAAGGAGCCCAATTCCA, targeting the hemagglutinin gene. We conducted real-time PCR for varicella zoster virus and herpesvirus using the ARGENE kit (bioMérieux, https://www.biomerieux-diagnostics.com). We ran PCR for DNA of the 16S RNA gene in parallel, adding to the specific PCR targeting *Bartonella* spp., *Francisella tularensis*, and *Rickettsia* spp. using primers previously reported (*24*,*25*).

For culture, we macerated the biopsy sample in Potter-Elvehjem PTFE tissue grinder (Dominique Dutscher Company, shttps://www.dutscher.com) and resuspended it in Hanks’ solution (Thermo Fisher Scientific, http://www.thermofisher.com). Afterward, we inoculated 200 μL of the sample containing 1 mL of Vero (ATCC CCL-81) African green monkey kidney cells at 10^6^ cells/mL onto each of 2 shell vials using 7 mL TRAC bottle (Thermo Fisher Scientific). We placed one at 32°C and the other at 37°C under 5% CO_2_ atmosphere and observed the vials daily under an inverted microscope to detect any potential cytopathic effect.

For virus production, we prepared 15 flasks of Vero cells in minimum essential medium (MEM) (Thermo Fisher Scientific) with 5% of fetal bovine serum and 1% of glutamine. After the cells reached 80% confluence, we removed the medium and inoculated the monolayer with 5 mL of viral suspension with a multiplicity of infection of 0.01. We incubated the flasks at 37°C for 1 hour for adsorption, then added 20 mL of modified MEM to the flasks and incubated them for 3 days. On the third day, we discarded the supernatant, then washed the cell monolayer 3 times with phosphate buffered saline and removed it using a scraper. After all the flasks were scraped and washed twice to collect the cells, we transferred the contents to 50-mL falcon tubes that were kept on ice.

We then centrifuged the cells at 1,500 rpm for 10 min, discarded the supernatant, and re-suspended the pellet in 10 mL of a sterile lysis buffer (MgCl_2_ 1 mmol/L, Tris 10 mmol/L, pH 7.0 KCl 10 mmol/L). We incubated the suspension for 10 min on ice. We performed mechanical lysis using a sterile tissue grinder (Dominique Dutscher Company, https://www.dutscher.com) (80 cycles on ice). We added 10 mL of 36% sucrose to a plastic centrifugation tube and transferred the viral mixture slowly, avoiding mixing with the sucrose solution (biphasic final solution). We centrifuged the tube at 14,000 rpm for 1 h at 4°C, collected the pellet, and stored it at −80°C in small aliquots.

### Micrograph Embedding and Cell Preparation for the Replicative Cycle

Hep2 cells (ATCC accession no. CCL-23) were maintained in culture with MEM modified with 10% of fetal bovine serum. The virus inoculated the Hep2 cell monolayer at a multiplicity of infection of 0.01. We then collected the content after scraping the flask at 32 h postinfection. We followed the same protocol of cell embedding as described by Bou Khalil et al. (*26*), except that we replaced the Epon resin with LR white resin (Agar Scientific, https://www.agarscientific.com). In brief, we fixed cells for 1 h with 2.5% glutaraldehyde in a 0.1 mmol/L sodium cacodylate buffer and washed them with a mixture of 0.2 mmol/L saccharose and 0.1 mmol/L sodium cacodylate. Postfix was for 1 h with 1% OsO4 diluted in 0.2 mmol/L potassium hexa-cyanoferrate (III) and 0.1 mmol/L sodium cacodylate solution. After washing with distilled water, we gradually dehydrated the cells with ethanol, and then gradually replaced the ethanol with LR white resin. We performed polymerization for 24 h at 60°C. We used a UC7 ultramicrotome (Leica) to obtain ultrathin 70-nm sections and placed them onto HR25 300 mesh copper/rhodium grids (TAAB Laboratories Equipment Ltd., https://www.taab.co.uk). We colored sections with Reynolds solution and obtained electron micrographs on a Tecnai G2 TEM (FEI, https://www.fei.com) operated at 200 keV. We used ImageJ software (https://imagej.nih.gov/ij) to determine particle size.

### Genome Sequencing and Assembling

We sequenced genomic cowpox virus DNA (DNAg) on MiSeq technology (Illumina Inc., https://www.illumina.com) with the paired end strategy and barcoded samples to be mixed with 18 other genomic projects prepared with the Nextera XT DNA sample prep kit (Illumina). We quantified the DNAg by high-sensitivity Qubit assay (Life Technologies, https://www.thermofisher.com) to 0.5 ng/µL and performed dilution requiring 1 ng of each genome as input to prepare the paired end library. The tagmentation step fragmented and tagged the DNA. Twelve cycles of limited-cycle PCR amplification completed the tag adapters and introduced dual-index barcodes. After purification on AMPure XP beads (Beckman Coulter Inc., https://www.beckman.com), we normalized the libraries on specific beads in accordance with the Nextera XT protocol (Illumina). We pooled normalized libraries into a single library for sequencing on the MiSeq, then loaded the pooled single-strand library onto the reagent cartridge and then onto the instrument, along with the flow cell. We performed automated cluster generation and paired-end sequencing with dual index reads in a single 39-hour run in 2 × 250-bp.

We obtained total information of 4.3 Gb from a cluster density of 343,000/mm^2^ with a cluster passing quality control filters of 97.8% (8,331,000 clusters). Within this run, we determined the index representation for cowpox virus to be 9.74%. We filtered the 811,395 paired-end reads according to the read qualities.

We assembled paired-end reads by using CLC genomics workbench version 7.5 (https://www.clcbio.com/) using 64-world size. The genome’s extremities appeared incomplete in comparison to the reference strains. Mapping against cowpox virus France Nancy 2001 (GenBank accession no. HQ420894.1) as reference showed a missing part in the 2 ITRs. We completed the inverted terminal repeat (ITR) regions by PCR followed by sequencing using primers previously designed on primer-Blast (*27*).

### Gene Prediction and Analysis

We computed gene prediction using Genemarks (*28*) and confirmed by Prodigal (*29*). We realized a blastp (https://blast.ncbi.nlm.nih.gov/Blast.cgi?PAGE=Proteins) of all predicted proteins against the nonredundant database. To determine average nucleotide value, we compared close phylogenetic strains using the ANI online calculator (https://www.ezbiocloud.net/tools/ani) based on the OrthoANI algorithm (*30*). Proteinortho (*31*) was used to determine best reciprocal hits using coverage of 80%, identity 20%, and an E-value cutoff established at 0.01. The genome sequenced in this study is available on the EMBLD/EBI website (accession no. LT883663).

### Phylogenetic Analysis

We computed alignments using MAFFT version 7 (*32*) with fast Fourier transform, a heuristic progressive method (FFT-NS2), on a 73-nt complete genome obtained from the Virus Pathogen Database and Analysis Resource (https://www.viprbrc.org/brc/home.spg). Alignments were manually controlled on MEGA version 6.0 (*33*).We used the FastTree program (*34*) to construct a maximum-likelihood tree using standard parameters with the Jukes-Cantors method for the nucleotide distances calculation with 1,000 local resamples (Shimodaira-Hasegawa test). We visualized trees by using iTol (*35*).

## Results

### Isolation of the Cowpox Virus and Clinical Context

 All bacterial PCR were negative and excluded any DNA bacterial presence. However, specific orthopoxvirus PCR was positive. In parallel, the inoculation on Vero cell showed a typical cytopathic effect after 4 days at 32°C and 37°C. Because cowpox is a notifiable disease, we reported the case to government authorities.

All vaccinations for the patient were up to date; he had received an injection against smallpox with vaccinia virus strain Lister when he was 1 year of age. Following governmental recommendations, no booster vaccination was given after the first injection. The patient did not report other chronic diseases, allergies, or addictions. He reported having a domestic cat at home who also lived outside. The patient’s cat was examined by a veterinarian and showed no sign of cowpox infection during this period. The patient is sure he was not scratched by his cat before the work accident occurred.

### Cowpox Virus Strain Genomic Analysis

We obtained a 219,385-bp genome with a GC content estimated at 33.6%. The gene prediction established the number of open reading frames at 214. Altogether, 212/214 predicted proteins had results in the nonredundant database; most (191) best-hit results were obtained for cowpox virus from various previously described strains, 9 for vaccinia virus, 4 for variola virus, 3 for monkeypoxvirus, 2 for ectromelia virus, and 1 each for horsepox, camelpox, and taterapox. The 2 other genes were considered as ORFan (Open Reading Frames without detectable homologues in other lineages), located in the ITR regions. We decided to explore the phylogeny of this new isolate. 

**Figure 2:**
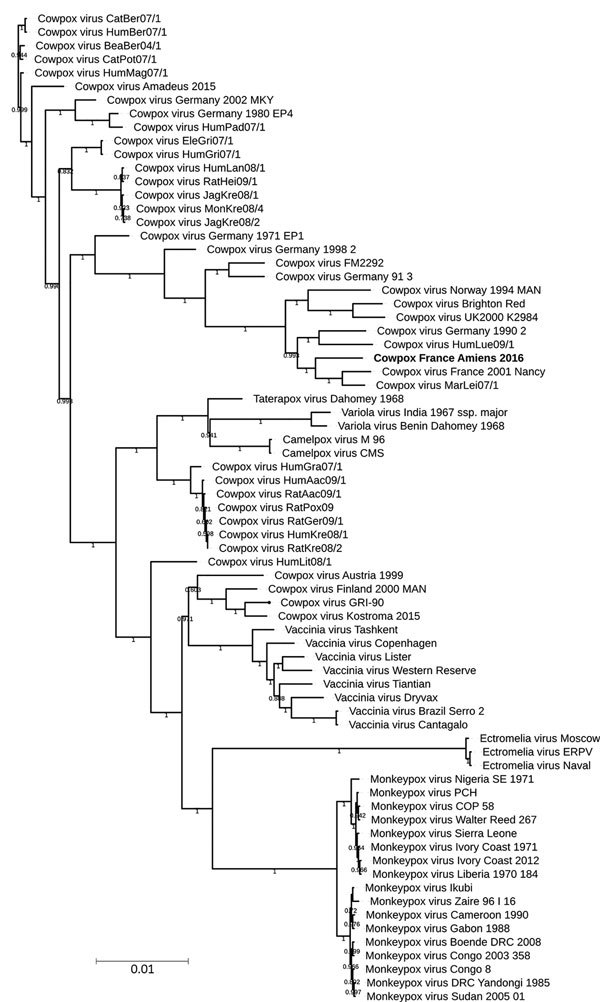
Phylogenetic tree of 73 orthopoxviruses, including cowpox virus isolate obtained from smallpox-vaccinated patient in France, 2016 (boldface). The tree includes data from 162,829 positions on central regions. Branches with a bootstrap value below 0.5 were deleted. Numbers shown on branches indicate bootstrap scores (e.g, 1.0 represents 100%). Scale bar indicates nucleotide substitutions per site.

A central part of the orthopoxvirus genome is extremely conserved. Regarding the recent proposed classifying elements of cowpox virus (*12*,*13*,*36*,*37*), we performed phylogenetic analysis on the available whole genome. We observed a subtype containing the novel strain, cowpox virus France Amiens 2016, along with the Nancy 2001 strain, the MarLei07/1, the HumLue09/1, and the Germany 1990 strains (Figure 2). Using the OrthoANI algorithm, we observed that France Amiens 2016 presented the highest similarity, 98.54%, with the cowpox France Nancy 2001 virus (Appendix Table 1). Moreover, the amino acid comparison of the main functional proteins showed a clear difference between the reference cowpox virus Brighton red strain and the other reported strains of the same cluster (Appendix Table 2). Taking all of these elements into consideration, we believe that cowpox virus France Amiens 2016 represents a new original strain clustering with the proposed E3 subclade in Europe (*12*).

**Figure 3:**
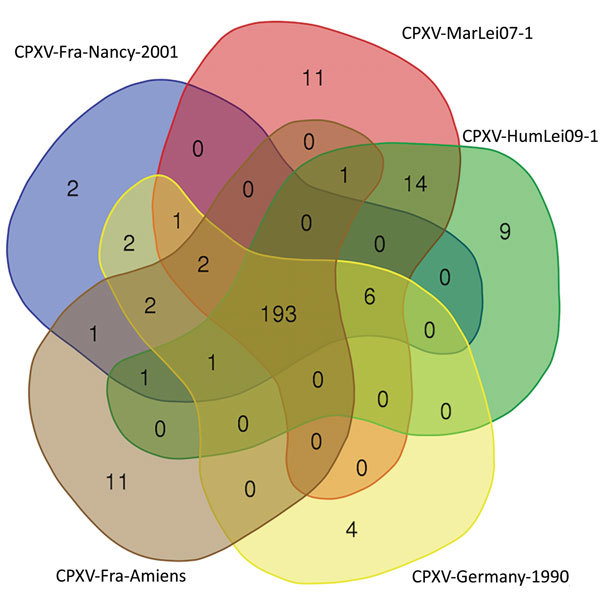
Venn diagram of reciprocal best hit obtained in the CPXV subclade E3, including the isolate obtained from a smallpox-vaccinated patient in France in 2016 (CPXV-Fra-Amiens). Diagram created by using the Bioinformatics & Evolutionary Genomics visualization tool (https://bioinformatics.psb.ugent.be/webtools/Venn). CPXV, cowpox virus.

Among the orthopoxvirus genus, the genomes’ evolution seems to be driven by numerous deletions affecting the number of predicted proteins, which could lead to a reduction of the genome length (*38*). To investigate predicted proteins in this group, we defined the cluster of orthologous proteins by reciprocal best hit. Five genomes of the defined E3 clade shared 193 predicted proteins (Figure 3). For the other clusters, we detected only duplicate proteins and variations in the size of the proteins by modifications of the start codon or by the modification of the stop codon (data not shown).

**Figure 4:**
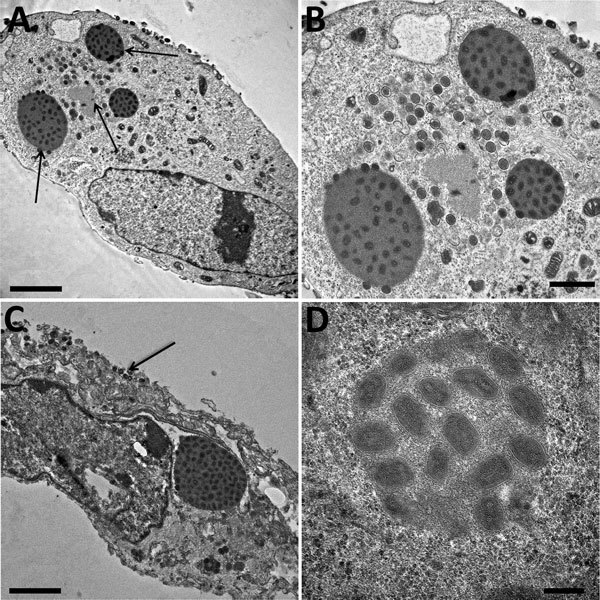
Electron microscopy imaging of cowpox virus France Amiens 2016, obtained from a smallpox-vaccinated patient in France in 2016. A) Ultrathin sections of a Hep2 cell at 32 hours postinfection. The cell harbors, which is undergoing its replicative cycle. Arrows indicate dense inclusion bodies as well as its viral factory containing viral crescents in the cell cytoplasm. Scale bar indicates 2 μm. B) Higher magnification of Hep2 cell in panel A; scale bar indicates 1 μm. C) Ultrathin sections of a Hep2 cell with a typical inclusion of cowpox virus detected near the nucleus. Arrow indicates extracellular-enveloped viruses or cell-associated enveloped particles. Scale bar indicates 2 μm. D) Electron-dense inclusion body containing mature viral particles. Scale bars indicate 200 nm.

To complete the description of this new isolate, we explored the morphological features in the viral replicative stage. Electron microscopy showed a typical A type inclusion (Figure 4) in the cytoplasm, classifying the cowpox France Amiens 2016 virus in the V+ subtype (*39*).

## Discussion

The story of orthopoxviruses seems to be clear, but many clues are still missing and ambiguous, where the literature shows much divergent data regarding traced sources, reservoirs, and contamination routes and tools. We are aware that rats, mice, raccoons, and field and bank voles are all recognized as susceptible to cowpox virus, and some of these animals can be associated with human cowpox virus infections. Moreover, serologic evidence highlights wide cowpox virus distribution in rodents and in cats (*40*,*41*). Bovids were considered a reservoir before the studies of Baxby (*14**,**42*) hypothesized that infections in cows and humans occurred when contaminated brambles and barbed wire were in the proximity of the cattle, but no data confirmed this last assertion. Nevertheless, it is important to highlight that transmission of other orthopoxviruses by fomites is well documented, especially for vaccinia virus, which can be transmitted among cattle by milking devices, as in Brazil (*43*).

This transmission by fomites should be integrated into upcoming studies with the availability of improved tools, notably in molecular biology, cell culture, and genome sequencing. Nevertheless, the case we report highlights that cowpox virus infection can be misdiagnosed by an atypical clinical presentation, resembling varicella zoster lesions or even noninfectious related rashes. In addition, for this case, it was not possible to establish an epidemiologic link between the patient and the typical sources of infection. Because the tool that caused the wound was kept on the ground, it is possible that it had been contaminated by contact with rodent or cat urine or feces. As is the case in almost all reported infection cases where the diagnosis occurs months later, it becomes difficult to retrace and investigate the route, initial host, or reservoir at an early time of infection. Another scenario is contact between patient and cat after the patient injury, something that was not reported by the patient, but this second potential route of infection appeared doubtful when we examined the co-localization between the injury with the guardrail and the eschar.

Smallpox vaccination is known to confer cross-immunity against other orthopoxviruses (*44**,**45*) with a high rate of success when the injection was done in preexposure conditions compared with postexposition. However, despite the patient’s smallpox vaccination, novel infections by orthopoxvirus (*46*) could have occurred. This cowpox infection is the result of a nonprotective status for the patient; possible causes include an absence of cross-reaction between vaccinia strain and cowpox virus subclade E3 or too long a period between immunization and exposition (nearly 44 years). We have no access to serologic tests or other analyses that were performed before the infection that could be used to confirm one of these hypotheses over another.

Finally, this case is the third reported in Europe within a year (*10*,*47*). As confirmed by genomic comparison in some geographic clusters, various strains seem to be circulating in wildlife in Europe, which is alarming because the diagnosis is always delayed when orthopoxvirus infection is not an initial suspect. In this context, the emergence and reemergence of diverse strains of orthopoxvirus must be seriously taken into consideration (*48*), as should the lack of investigation of potential outbreaks. Multiplying new genome sequences associated with exhaustive clinical reports seems to be an appropriate strategy (*12*,*49*) to explore cowpox virus diversity and variants in Europe in general and France in particular.

Appendix**.** Additional information from the analysis of cowpox infection in a patient who had been vaccinated for smallpox.
